# Wafer-Scale Patterning of Protein Templates for Hydrogel Fabrication

**DOI:** 10.3390/mi12111386

**Published:** 2021-11-12

**Authors:** Anna A. Kim, Erica A. Castillo, Kerry V. Lane, Gabriela V. Torres, Orlando Chirikian, Robin E. Wilson, Sydney A. Lance, Gaspard Pardon, Beth L. Pruitt

**Affiliations:** 1 Department of Materials Science and Engineering, Uppsala University, 751 03 Uppsala, Sweden; anna.kim@angstrom.uu.se; 2 Department of Mechanical Engineering, University of California, Santa Barbara, CA 93106, USA; ericasti@stanford.edu (E.A.C.); klane@ucsb.edu (K.V.L.); gvillalpandotorres@ucsb.edu (G.V.T.); 3 Department of Mechanical Engineering, Stanford University, Stanford, CA 94305, USA; rew318@gmail.com (R.E.W.); sydneyannlance@gmail.com (S.A.L.); 4 Biomolecular Science and Engineering Program, University of California, Santa Barbara, CA 93106, USA; ochirikian@ucsb.edu; 5 Department of Bioengineering, School of Engineering and School of Medicine, Stanford University, Stanford, CA 94305, USA; gaspard@stanford.edu; 6 Department of Molecular, Cellular and Developmental Biology, University of California, Santa Barbara, CA 93117, USA

**Keywords:** microfabrication, lift-off protein patterning, hydrogels, single-cell cardiomyocytes, single-cell analysis

## Abstract

Human-induced pluripotent stem cell-derived cardiomyocytes are a potentially unlimited cell source and promising patient-specific in vitro model of cardiac diseases. Yet, these cells are limited by immaturity and population heterogeneity. Current in vitro studies aiming at better understanding of the mechanical and chemical cues in the microenvironment that drive cellular maturation involve deformable materials and precise manipulation of the microenvironment with, for example, micropatterns. Such microenvironment manipulation most often involves microfabrication protocols which are time-consuming, require cleanroom facilities and photolithography expertise. Here, we present a method to increase the scale of the fabrication pipeline, thereby enabling large-batch generation of shelf-stable microenvironment protein templates on glass chips. This decreases fabrication time and allows for more flexibility in the subsequent steps, for example, in tuning the material properties and the selection of extracellular matrix or cell proteins. Further, the fabrication of deformable hydrogels has been optimized for compatibility with these templates, in addition to the templates being able to be used to acquire protein patterns directly on the glass chips. With our approach, we have successfully controlled the shapes of cardiomyocytes seeded on Matrigel-patterned hydrogels.

## 1. Introduction

Human-induced pluripotent stem cell-derived cardiomyocytes (hiPSC-CMs) have gained significant traction over the last decade as a powerful model for understanding cardiac development, modeling cardiac diseases, drug screening, and cardiotoxicity screening [1]. hiPSCs have become more widely used because primary adult cardiomyocytes (CMs) do not regenerate and present difficulty when creating in vitro cultures [2]. hiPSC-CMs are derived from patient somatic cells, reprogrammed to a pluripotent state, and then differentiated into cardiomyocytes [3]. They hold great promise for personalized medicine and can be genetically edited to display various mutations linked to diseases, making them an attractive model [4]. Despite the potential of hiPSC-CMs as powerful models, they are limited by the immaturity and heterogeneity that is observed not only across different lab groups and lab members, but also across batches, even when using the same protocols [1]. hiPSC-CMs display a fetal-like phenotype in terms of a sarcomere structure, t-tubule organization, metabolism, calcium handling and overall morphology [5]. Current methods to improve CM maturity include a prolonged culture time, the addition of biochemical cues, biophysical stimulation, altering substrate stiffness and/or extracellular matrix (ECM) proteins [5,6]. The in vitro microenvironment can have a drastic effect on hiPSC-CM maturation and promote a more adult, rod-like CM structure and organized sarcomeres [6]. Amongst the most common methods currently used to culture hiPSC-CMs in vitro, one features a monolayer of CMs cultured on polystyrene tissue culture plastic that has been physisorbed with ECM proteins such as laminin, fibronectin, collagen, or Matrigel [7,8,9].

Tunable micropatterned protein platforms for cell cultures are becoming widely used to manipulate cells because they can control cellular spatial organization and mimic properties of the local microenvironment with a reductionist order approach [10]. The ability to manipulate the in vitro microenvironment and to provide physiologically relevant cues is important for the development of the mechanobiology field as cells are known to sense their local environment, leading to changes in gene transcription, morphology (i.e., cell shape, internal cell organization and cell and tissue architecture) and function (i.e., migration, division and differentiation) [11]. In the case of cardiomyocytes, tunable hydrogel devices are promising because they can also recapitulate the native mechanical microenvironment properties [6]. Hydrogels are composed of a polymer network swollen with water, allowing for inclusion of micropatterns of specific cell adhesion ligands [12,13]. They are highly tunable in terms of their mechanical stiffness, pore size and swelling based on the polymer type, pre-polymer concentration and crosslinking density.

Studies have shown that the substrate stiffness and extracellular matrix components can modulate the cardiomyocyte contractility, cytoskeleton structure, differentiation lineage and adhesion area [14,15,16,17] (reviewed in [18]). Furthermore, protein micropatterning platforms reduce the cell population heterogeneity by constraining the cell shape, which allows for easier cell normalization [19]. Assessing the functional contractility of CMs is important for understanding the relationship between cell structure and function. These hydrogel platforms allow for fiducial microbeads to be embedded into the platform, enabling functional contractility measurements, such as traction force microscopy [20,21,22]. Other methods for assessing changes in the active forces that CMs generate have been reviewed in [23].

Native CM cytoskeleton structure, anisotropic contraction direction, and contractility have been recapitulated by manipulating the microenvironment. One study found that neonatal rat ventricular myocytes cultured on rectangular extracellular matrix (ECM) patterns of various aspect ratios aligned their sarcomeres in predictable and repeatable patterns, which is in contrast to circular myocytes [24]. hiPSC-CMs with 7:1 aspect ratio (length by width) protein patterns had increased myofibril alignment and contractile force output when compared to smaller pattern aspect ratios (3:1, 1:1 and non-patterned) [21]. The protein micropatterning platforms can yield cells that adhere in known spacing intervals, which is ideal for image acquisition and can be aligned with high throughput screens [25,26,27,28].

Many methods to yield protein-micropatterned hydrogels exist [12]; however, these methods often require cleanroom facilities and microfabrication expertise. Additionally, current technology is often made serially which results in a slow fabrication workflow. Lastly, the challenge of obtaining reproducible and high-quality protein patterns remains [29]. To study cell–ECM and cell–cell protein interactions and spatially confine cells, cell culture substrates may be functionalized with proteins of interest using micropatterning techniques. Microcontact printing (µCP) is a commonly used technique for protein micropatterning on both soft and rigid substrates. The technique utilizes a flexible microfabricated stamp that is inked with a protein and put in contact with a cell culture platform to transfer the protein pattern.

Many groups have used µCP because the protocols are straightforward and widely accessible; however, the technique is limited by the resulting pattern accuracy and resolution [30]. To increase throughput, it is possible to generate protein patterns over large areas on glass by selectively oxidizing biopassive poly(l-lysine)-grant-poly(ethylene glycol) (PLL-g-PEG) copolymers and backfilling exposed regions with a protein [25,30,31,32]; however, most methods involving PLL-g-PEG require microfabrication equipment that is not commonly available in many laboratories. We have recently established a photoresist lift-off patterning method that is more reproducible than µCP [30]. This method has created higher fidelity patterns and allowed for storage of the photoresist protein templates; however, the method relies on a serial process, and hence it is time-consuming to generate many individual microscopy coverslips and requires working in specialized microfabrication facilities.

Here, we present a batch wafer-scale approach for the photoresist lift-off patterning method that (1) generates a high-yield of glass chips (16 chips per 4” wafer) for (2) protein patterns with high reproducibility and accuracy with (3) long shelf stability. This batch method results in photoresist protein templates on glass chips that can be used to either make protein-patterned hydrogels or protein patterns directly on the chips. We scaled up the photolithography processing step, since this part of the lift-off protocol was one of the main bottlenecks and still allows for a high degree of flexibility in the design and subsequent fabrication of protein-patterned hydrogels, for example, tuning the mechanical properties of hydrogels (e.g., stiffness), and the selection of extracellular matrix proteins occurs at a later stage in the protocol [33]. In addition, the original photolithography step was the most time-intensive part of fabrication since each template was created serially.

Importantly, our wafer-scale method can be used to generate a large quantity of pattern templates that can be used for more than six months after wafer fabrication and dicing. The shelf stability of the photoresist-patterned glass chips makes it possible to externally source the pattern templates and thus removes the requirement from labs to have cleanroom infrastructure and expertise. The remaining steps in the fabrication of hydrogels do not require specialized equipment besides a chemical fume hood. Further, we have integrated spacers into the hydrogel fabrication method for precise and uniform control of the final hydrogel thickness, which is an important parameter for high-resolution microscopy. Here, we demonstrate the transfer of protein patterns onto hydrogels for a wide range of cell culture substrates, ranging from multi-well cell culture plates to coverslips. Finally, we characterize the performance of Matrigel-patterned hydrogels by demonstrating how single-cell hiPSC-CMs adhere, spread to a high aspect ratio (above 3:1) and actively contract on the hydrogels.

## 2. Materials and Methods

The final hydrogels used for cell-seeding and imaging were protein-patterned hydrogels with a stiffness of 10 kPa, adhered to a cell culture substrate, in our case, a glass-bottom 6-well plate. The protein patterns inform protein interactions for the cells within a defined space; we designed the patterns to study single hiPSC-derived cardiomyocytes. The protein patterns had an area of 1500 µm^2^ with an aspect ratio of 7:1 (length by width), which helps guide the alignment of myofibrils, thus facilitating a more mature cell phenotype. The single-cell patterns were spaced at intervals of 50 µm along the x- and y-axis, filling the entire 5 × 7 inch mask so as not to require alignment during the dicing process. This section describes the fabrication process of the hydrogels, including the (1) wafer fabrication process on 4” glass wafers using photolithography and dicing to obtain individual chips and (2) the development and (3) transfer of protein patterns to hydrogels using lift-off and copolymerization techniques.

### 2.1. Wafer Fabrication Process and Dicing

**Figure 1:**
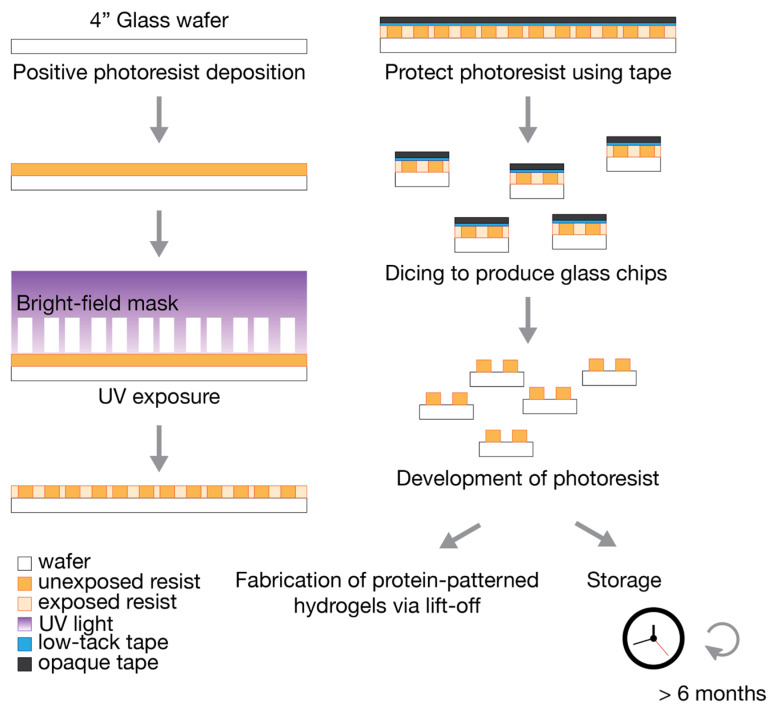
Wafer fabrication of 4” glass wafers using photolithography, from photoresist deposition to dicing to obtain individual glass chips, and development of the photoresist. Developed glass chips can be stored for at least six months in a light-protected environment prior to the fabrication of protein-patterned hydrogels.

To scale up the photolithography process (summarized in Figure 1), we selected 4” glass wafers for their similarity in surface properties to the currently used glass microscopy coverslips [30] (e.g., 48382-085, VWR). We chose 500-µm-thick D263 glass wafers (1617, University Wafer, Boston, MA, USA) due to their low cost and robustness. Thinner glass wafers can also be used. We tested 200-µm-thick borosilicate glass wafers (2248, University Wafer); however, they were significantly more delicate to handle and release from the dicing tape.

The 4” glass wafers were thoroughly cleaned with acetone, followed by isopropanol, and then deionized water. Plasma treatment is not recommended as it changes the surface properties of the material, and we observed that this treatment could lead to detachment of the photoresist at the development step. The wafers were dried using a flow of nitrogen gas and then dehydrated on a hotplate for 5 min at 180 °C. Positive photoresist AZ1512 (Merck Performance Materials, Merck KGaA, Darmstadt, Germany) was spun first at 500 rpm for 10 s and then ramped up to 2000 rpm for 45 s in order to achieve a 2-µm-thick resist layer. A soft bake was performed with a level hotplate for 2 min at 100 °C. The photoresist was exposed (Karl Suss MA6 aligner, SÜSS MicroTec, Garching, Germany) to achieve 50 mJ/cm^2^ at 365 nm using a bright-field mask for transparency (CAD/Art Services, Bandon, OR, USA). The exposure time was based on a daily calibration of the light source using a power meter. For example, when the power meter measured 9 mW/cm^2^, the exposure time was adjusted to 5.6 s. For exposure, we used soft or hard contact modes to extend the mask lifetime.

Low-tack surface protection tape (6317A18, McMaster-Carr, Elmhurst, IL, USA) was gently applied on the photoresist-covered wafer, followed by cleanroom masking tape (76505A8, McMaster-Carr) to protect the photoresist from further exposure to light. Excess tape was cut away with a microtome blade. The tape-covered wafers were diced using a dicing saw (ADT 7100, Advanced Dicing Technologies Ltd., Zhengzhou, China) with a thermocarbon diamond blade (2.817-4C-30R-3, Thermocarbon Inc., Casselberry, FL, USA) at a spindle speed of 25,000 rpm, a cut speed of 5 mm/s and a reduced cut water pressure of 0.6 splm to reduce tape delamination. The 4” glass wafer was cut 7 × 7 times at 0° and 90° angles. The dimensions of each glass chip were 15 mm × 15 mm, yielding more than 16 chips per wafer.

### 2.2. Development

The glass wafer was attached to the dicing fixture with ultraviolet (UV)-release tape. Since the wafer is transparent with photoresist patterns, we did not use UV light to release the tape. Instead, the chips were carefully peeled away from the tape and the photoresist AZ1512 was developed in AZ 300 MIF (Merck Performance Materials, Merck KGaA, Darmstadt, Germany) for 60 s and rinsed with distilled water. Several chips were developed at the same time using a mini-rack holder (Z688568, Merck KGaA, Darmstadt, Germany). Diced glass chips with a developed photoresist can be stored in a light-protected environment for more than six months prior to lift-off protein patterning and hydrogel fabrication.

### 2.3. Fabrication of Hydrogels with Protein Patterns

The transfer of protein patterns to hydrogels using lift-off is described in detail in [30]. Briefly, glass chips with developed photoresist patterns were incubated with PLL-g-PEG (SuSoS, Dübendorf, Switzerland) for 60 min at 100 μg/mL. The remaining photoresist was lifted off using varying concentrations of N-methyl-2-pyrrolidone (NMP, Merck Performance Materials, Merck KGaA, Darmstadt, Germany) in MilliQ water (Milli-Q, MilliPoreSigma, Merck KGaA, Darmstadt, Germany). The glass chips were first submerged in a mixture of ⅔ MilliQ, ⅓ NMP for 20 s, then pure MilliQ water for 10 s. The glass chips were then submerged and sonicated in pure NMP for 6 min, then submerged and sonicated in a mixture of ½ MilliQ, ½ NMP for 1 min. Finally, the chips were rinsed in fresh MilliQ water for 5 min before we incubated the protein of interest on them. We used fluorescent labeled gelatin (G13186, Thermo Fisher Scientific, Waltham, MA, USA) to visualize the transferred protein patterns on the hydrogels, which we incubated on the glass chips for 60 min at room temperature. For hydrogels that were seeded with hiPSC-CMs, we used Matrigel (356252, Corning, Corning, NY, USA) as the ECM protein at a concentration of about 1000 μg/mL, which we incubated on the glass chips for 1 h at room temperature.

The polyacrylamide hydrogel was adhered by chemically treating the glass coverslip or glass well plate with bind-silane. Briefly, the bind-silane solution (3 µL bind-silane, 50 µL acetic acid and 950 µL 95% ethanol) was prepared in a chemical fume hood. The bind-silane was purchased from Sigma (3-(trimethoxysilyl) propyl methacrylate (M6514, Merck KGaA, Darmstadt, Germany). Next, the glass was treated with oxygen plasma for 15 s at 80 W or at a high setting. Immediately following plasma, ~50 μL of the bind-silane mixture was added to cover the entire glass substrate. After reacting for 1 min, the excess bind-silane was removed and the remaining solution was allowed to react for 10 min. Finally, the glass substrates were rinsed twice with 1 mL of ethanol, dried with nitrogen gas and allowed to dry in a desiccator until ready for use.

After protein incubation, polyacrylamide (PA) precursor solutions were prepared for casting the hydrogels using a previously published protocol with slight adjustments [34]. Briefly, we prepared 0.5 g/mL acrylamide (01696, Merck KGaA, Darmstadt, Germany) and 0.025 g/mL bis-acrylamide (146072, Merck KGaA, Darmstadt, Germany) solutions in MilliQ water. We combined 198 μL of the acrylamide solution and 40 μL of the bis-acrylamide solution, following the formulation for 10% T and 1% C hydrogels [34]. We added 21.6 μL of red fluorescent microbeads (F8812, Thermo Fisher Scientific, Waltham, MA, USA), a necessary element for traction force microscopy analysis, along with 140.5 μL of 250 mM HEPES buffer (N-2-hydroxyethylpiperazine-N-2-ethane sulfonic acid, 15630080, Thermo Fisher Scientific). We adjusted the volume of MilliQ water to 594.4 μL to account for the added volume of fluorescent microbeads and HEPES buffer. Separately, we prepared a 10% weight/volume solution of ammonium persulfate (APS, A9164, Merck KGaA, Darmstadt, Germany) in MilliQ water. We degassed the PA precursor solutions and the APS solution in a vacuum desiccator for 1 h.

**Figure 2:**
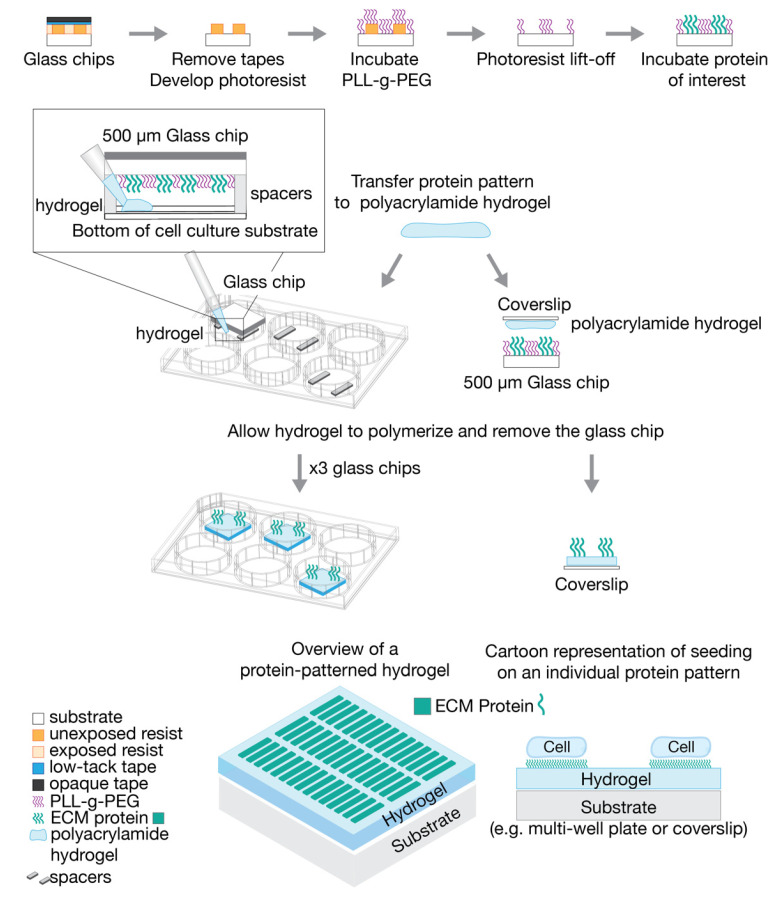
Protocol for generating protein patterns on diced glass chips from photoresist templates by lift-off and subsequent fabrication of protein-patterned hydrogels with protein patterns by transfer method.

To prepare for casting the hydrogels, 250-μm-thick polydimethylsiloxane (PDMS) spacers were introduced to define hydrogel thickness and make the hydrogel fabrication method compatible with different types of cell culture substrates when using 500 µm diced glass chips. Spacers are not needed when using glass microscopy coverslips due to the difference in weight. Figure 2 outlines the process for fabricating protein-patterned hydrogels with diced glass chips and microscopy coverslips. For diced glass chips, PDMS spacers were placed in the well of a glass-bottom 6-well plate (P06-1.5H-N, Cellvis, Mountainview, CA, USA). The patterned glass chip was then placed on top of the PDMS spacers, with the patterned side of the glass facing downward. As the hydrogel polymerizes, the ECM protein pattern is transferred and anchored to the hydrogel via the copolymerization physisorption method [20,35].

To begin polymerization, 5 μL of the 10% APS solution and 0.5 μL of N,N,N′,N′-tetramethylethylenediamine (TEMED, 411019, Merck KGaA) were added to the precursor solution. The solution was carefully mixed with a pipette, ensuring air bubbles were not introduced to the solution. For the diced glass chips, the solution was pipetted between the PDMS spacers until the solution spread throughout the entire sandwich, approximately 60 μL of solution total. For microscopy coverslips, 50 μL of the hydrogel solution was pipetted onto the cell culture substrate, then the coverslip was placed on top of the hydrogel solution, patterned side down. Following casting, the hydrogels were protected from light and left for 30 min to begin polymerization. After 30 min, the hydrogels were hydrated with phosphate buffered saline (PBS, 10010049, Thermo Fisher Scientific) and left to polymerize further at 4 °C for 6–8 h. After full polymerization, the diced glass chips and microscopy coverslips were removed from the hydrogels and discarded.

It is important to note that hydrogels are not shelf stable [34] and should be stored in a buffer solution. We recommend that cells are seeded on hydrogels within 72 h of full polymerization.

### 2.4. Maintenance of Induced Pluripotent Stem Cells and hiPSC-Derived Cardiomyocytes

Human-induced pluripotent stem cells (hiPSCs), with GFP-labeled alpha actinin, were purchased from Coriell Institute (AICS-0075-085, Camden, NJ, USA). hiPSCs were propagated on tissue culture plates coated with Matrigel (356252, Corning) using feeder-free culture conditions in standard culturing environments consisting of 5% carbon dioxide at 37 °C. The Essential 8 Medium (Gibco, Thermo Fisher Scientific, Waltham, MA, USA) was changed daily and cells were passed using EDTA when confluency reached 80%. hiPSCs were differentiated into hiPSC-derived cardiomyocytes (hiPSC-CMs) using previously published methods [36]. Upon the initiation of beating (day 7–8), glucose starvation was utilized to purify hiPSC-CMs from other contaminating cell types. On day 12, we utilized the previously published expansion protocol [37] to propagate a significant number of hiPSC-CMs for the entirety of this study. After two passages of expansion treatment, hiPSC-CMs were lifted using EDTA and cryopreserved using xeno-free cryopreservation media Bambanker (Lymphotec, Tokyo, Japan) at a density of 1 million cells/mL. hiPSC-CMs were cooled at a rate of 1 °C per minute using a Nalgene Mr. Frosty in a −80 °C freezer for 24 h. The following day hiPSC-CM cryovials were transferred and remained in liquid nitrogen until thawed.

When protein-patterned hydrogels were ready for seeding, hiPSC-CMs were thawed for 2 min in a 37 °C water bath and centrifuged at 1000 rpm for 5 min. Subsequently, the cryopreservation medium was removed, the hiPSC-CMs were resuspended in replating media (RPMI supplemented with B27 + Thiazovivin (2 μM) + 10% KnockOut Serum Replacement Media) and then replated on our protein-patterned hydrogels at a final density of 250,000 cells.

### 2.5. Microscopy and Data Analysis

We verified that the hiPSC-CMs were adhered and beating on the Matrigel-patterned hydrogels (Video S1) at 4 days post-seeding. The cells were fixed in 4% paraformaldehyde diluted with PBS (10010049, Thermo Fisher Scientific) for 5 min and then rinsed three times with PBS and stored in PBS at 4°C.

Microscopy images were acquired with a Zeiss Axio Observer 7 inverted microscope and a Photometrics Prime 95b camera. For high magnification images, a 40× objective (Zeiss, Jena, Germany, LD Plan-Neofluar 0.6 NA) was used. Overviews of the entire protein-patterned hydrogels were acquired using Zeiss Zen 2.5 blue microscopy software together with ConTraX [28], which is a software developed in our lab for high-throughput single cell imaging and traction force measurement, for which a 10× objective (Zeiss, Jena, Germany, Plan Apochromat 0.45 NA) was used.

We applied the following morphology selection filter to analyze single hiPSC-CMs that took up an elongated aspect ratio within the ECM micropattern width and area. For the hiPSC-CM morphology data, we included the analysis for cells with a high aspect ratio (above 3:1). We note that it is possible to re-run the ConTraX cell morphology analysis with the same images and apply a different selection criteria if needed. For this reason, Excel File S1 contains the ConTraX data for all identified objects.

We performed further data selection and representation in Matlab 2019b (MathWorks, Natick, MA, USA) using the Statistics and Machine Learning toolbox. Identified objects with an area below 200 µm^2^ were discarded as debris. Stringent selection criteria only analyze high aspect ratio cells (between 3:1 and 9:1), and discard cells growing well outside the defined protein patterns (width > 16 µm) and likely cell doublets (area above 1900 µm^2^). Microscopy images were opened in Fiji [38] and illumination was pseudo-corrected when appropriate using the BioVoxxel toolbox [39].

## 3. Results

### 3.1. Wafer Fabrication and Protein Pattern Transfer to Hydrogels

**Figure 3:**
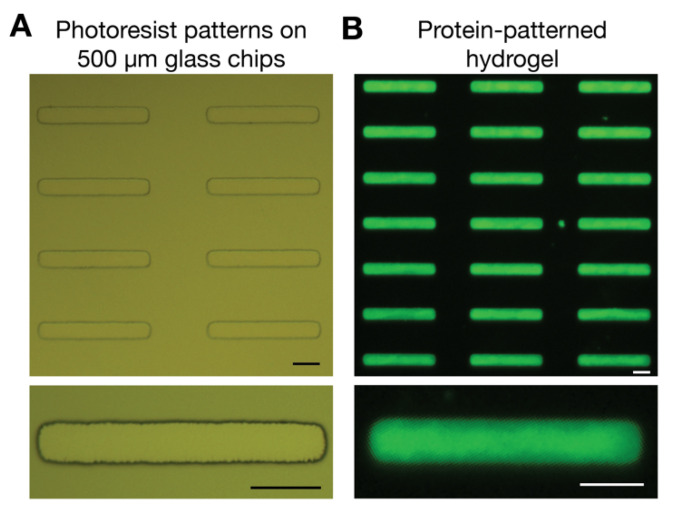
Transfer of protein templates in photoresist on glass chips into protein patterns on hydrogels. (**A**) Developed photoresist templates on glass chips. The developed photoresist templates were stored in a light-protected environment for approximately six months prior to hydrogel fabrication. (**B**) Protein patterns on hydrogels were visualized using fluorescent gelatin. Fluorescent gelatin was transferred from glass chips using polydimethylsiloxane (PDMS) spacers to define the hydrogel thickness. Scale bars denote 25 µm.

Wafer-scale fabrication of photoresist protein templates is a convenient and facile method for generating multiple glass chips to enable on-demand and consistent fabrication of protein-patterned hydrogels. This method makes it easy to make multiple hydrogels with different properties in terms of the stiffness or choice of extracellular matrix proteins. In addition, the glass chips are compatible with different types of cell culture substrates, from glass microscopy coverslips to multi-well plates. Figure 3 illustrates how the photoresist patterns (Figure 3A) translate into protein patterns on a hydrogel (Figure 3B) using fluorescent labeled gelatin. The diced glass chips with photoresist templates were stored for approximately six months prior to lift-off and protein pattern transfer to a hydrogel.

To obtain high-quality protein patterns on hydrogels, it is important to have photoresist patterns that are clean from debris and contaminations. For this reason, we developed the photoresist post-wafer dicing, but it then becomes critical that the photoresist is minimally exposed to light at all steps of the fabrication process. We used masking tape during dicing to ensure protection from light exposure. This light sensitivity limited our ability to release UV tape on glass wafers using UV exposure and so using thicker glass wafers, namely 500 µm here, significantly increased our yield of glass chips with photoresist patterns; however, thicker glass chips are also heavier than microscopy coverslips and we introduced spacers in the hydrogel fabrication method. The spacers serve three important purposes, where they (1) prevent the hydrogel from collapsing under the weight of the glass chips, (2) define the hydrogel thickness and (3) ensure even hydrogel thickness for high-resolution microscopy. This was critical to making the method compatible for different types of cell culture substrates when used with 500 µm glass chips and improving imaging capabilities.

The transfer of photoresist templates from glass coverslips to protein-patterned hydrogels has been characterized in [30]. We did not observe differences in quality among the glass chips, which are taken from the center of the wafer and exclude ~10% from the edges. We attributed this to the uniformity of the photoresist due to its thinness and processing (the use of a level hotplate); however, we did observe a decrease in quality of the transferred patterns close to the edges of the diced glass chips.

### 3.2. Single-Cell Cardiomyocytes on Protein-Patterned Hydrogels

**Figure 4:**
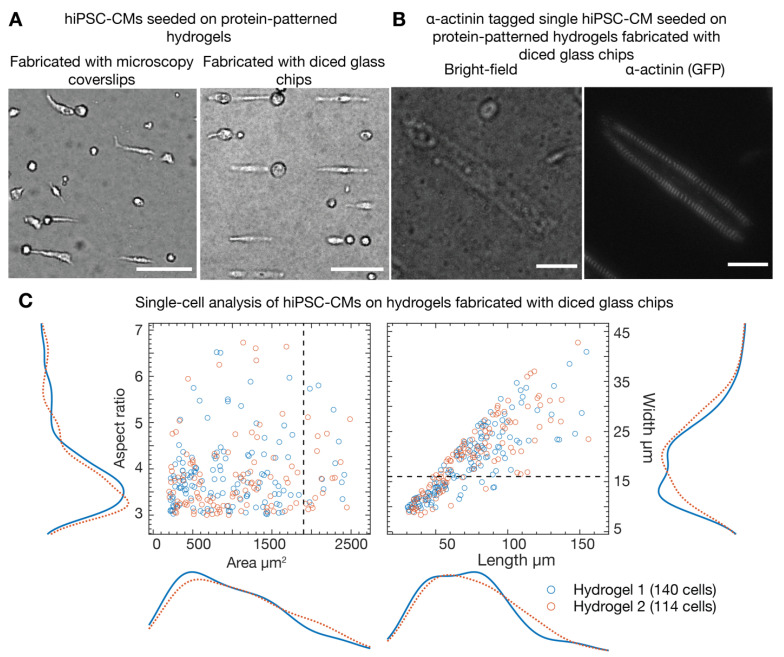
Human-induced pluripotent stem cell-derived cardiomyocytes (hiPSC-CMs) seeded on protein-patterned (Matrigel) hydrogels. (**A**) Comparison between cells seeded on hydrogels with protein transferred from microscopy coverslips fabricated in a serial process using the method described in [30] and from diced glass chips fabricated in a batch wafer-scale process as described in this paper. Scale bars denote 100 µm. (**B**,**C**) Analysis of cells on protein-patterned hydrogels fabricated from diced glass chips. (**B**) Microscopy images of an alpha-actinin labeled hiPSC-CM seeded on a hydrogel with protein transferred from a diced glass chip. Scale bars denote 25 µm. (**C**) Two-dimensional scatter plots of the aspect ratio and area and length and width, along with the marginal distributions of the parameters. The results are grouped by hydrogel with debris (objects with area below 200 µm^2^) filtered out and high aspect ratio cells selected (between 3:1 and 9:1). The criteria for cells that extend well beyond the protein patterns (width above 16 µm) and likely cell doublets (area above 1900 µm^2^) are indicated by the dashed lines. Cells were fixed for the analysis.

Expanded hiPSC-CMs were thawed and cultured for 10 days. The cardiomyocytes were seeded on single-cell Matrigel patterns on hydrogels with an aspect ratio of 7:1 and area of 1500 µm^2^. The cells were continuously monitored for their health and adhesion to the hydrogels. We verified that the cardiomyocytes were healthy and beating (Video S1) at 4 days after cell seeding onto the protein-patterned hydrogels. Furthermore, we compared the cell distributions for CMs seeded on protein-patterned hydrogels fabricated with (1) microscopy coverslips in a serial process described in [30] and (2) diced glass chips using the method reported in this paper. For comparison, we used a serial fabrication process [30] with the same single-cell protein templates (described in the Materials and Methods section) and seeded expanded cardiomyocytes. We found that the cell distribution on protein-patterned hydrogels using both fabrication methods was comparable (illustrated in Figure 4A). Protein-patterned hydrogels for these experiments were generated using diced glass chips with photoresist templates fabricated more than six months prior.

Using fluorescently-tagged alpha actinin cells, we have demonstrated the internal structure of high aspect ratio cardiomyocytes on protein-patterned hydrogels (Figure 4B). We studied how the cells adhered to the protein patterns by analyzing the distributions of cell area, aspect ratio, length, and width in fixed cells (Excel File S1). We have found that, per hydrogel from a 15 mm × 15 mm glass chip and assuming a usable area of 100 mm^2^ due to edge effects, >1000 cells adhere, occupying up to 15% of the total available micropatterns. Of these cells, ~120 cells (10%) adapted to the high aspect ratio (above 3:1) provided by the protein patterns (Figure 4C). Seeding at a higher cell density or growing cells on hydrogels for longer are potential strategies to increase the percentage of occupied patterns; however, this comes at the cost of an increased number of cell doublets and cells growing outside of the protein patterns. Already, we could see many cells growing well outside of the protein patterns (width above 16 µm) and likely cell doublets (area above 1900 µm^2^); however, even when using this stringent set of criteria, we typically obtained ~50 cells per hydrogel, which is sufficient for most high throughput experiments that would, in addition, use several hydrogels.

## 4. Discussion

In this work, we have presented a batch wafer-scale approach based on photolithography for lift-off protein patterning on polyacrylamide hydrogels that (1) generates a high-yield of glass chips for (2) protein patterns with high reproducibility and accuracy with (3) long shelf-life stability. A previous work has utilized individual small glass microscopy coverslips during the lithography stage [30]. This serial fabrication process ultimately results in a slow fabrication speed. In contrast, our wafer-scale approach creates many photoresist-patterned glass chips in parallel using a single wafer. We streamlined the photolithography part of the process to render the lift-off patterning method more accessible and scalable. Our work could be used as a roadmap to establish future collaboration with a cleanroom expertise team, in which wafer processing, lithography and dicing are common techniques. After the diced glass chips with photoresist patterns are made, the rest of the protocol is straightforward and can be performed with standard laboratory equipment. Furthermore, our work shows that the diced and developed photoresist glass chips can be stored for at least six months. This is significant since it allows for streamlined batch processing and decreases the required cleanroom time. This shelf-stability also allows for flexibility around cell culture maintenance.

Our work shows that lift-off patterning and the copolymerization transfer technique with polyacrylamide hydrogels is compatible with single cell hiPSC-CMs on Matrigel rectangular protein patterns. Previous work using lift-off protein patterning utilized Madin–Darby Canine Kidney (MDCK) cells on collagen I and gelatin protein patterns [30]. Furthermore, while we have only presented results with the polyacrylamide hydrogel formulation for 10 kPa here, previous work has also shown that lift-off is compatible with various hydrogel stiffness (e.g., 5, 10 and 25 kPa). Hence, our approach retains the previously demonstrated possibilities to work with varying cell types, single cell or multiple cells, ECM protein types, protein pattern geometries, and hydrogel stiffness. Additionally, since this platform has compatibility for live cell microscopy, other cell functional readouts can be easily added, such as traction force microscopy [21]. Our approach presented here can be used in future studies to increase our understanding of mechanobiology and how the microenvironment influences cell structure and function in both healthy and disease states.

## Data Availability

The data presented in this study are available within the article and Excel File S1.

## Supplementary Materials

The following are available online at https://www.mdpi.com/article/10.3390/mi12111386/s1, Video S1: recording of a single hiPSC-CM beating on a protein-patterned hydrogel; real time, Excel File S1: Area, aspect ratio, length, and width data of fixed single hiPSC-CMs adhered to protein-patterned (Matrigel) hydrogels. Hydrogels were scanned in entirety to obtain this data.
